# Factors Associated with Revision Sinus Surgery in Patients with Chronic Rhinosinusitis

**DOI:** 10.3390/jpm12020167

**Published:** 2022-01-27

**Authors:** Karina Bayer, Selmir Hamidovic, Gerold Besser, Christian A. Mueller, David T. Liu

**Affiliations:** Department of Otorhinolaryngology, Head and Neck Surgery, Medical University of Vienna, 1090 Vienna, Austria; karina.bayer@meduniwien.ac.at (K.B.); selmir.hamidovic@meduniwien.ac.at (S.H.); gerold.besser@meduniwien.ac.at (G.B.); david.liu@meduniwien.ac.at (D.T.L.)

**Keywords:** chronic rhinosinusitis, functional endoscopic sinus surgery, ESS, CRSsNP, CRSwNP, sinus surgery

## Abstract

Endoscopic sinus surgery (ESS) is performed in patients diagnosed with Chronic Rhinosinusitis (CRS) refractory to primary medical therapy to achieve adequate disease control. This study aimed to assess which factors and phenotypes of CRS are associated with revision surgery in patients undergoing ESS. This retrospective, single-center study included 667 patients undergoing ESS between 2012 and 2015. We performed group comparisons to detect differences between CRS patients undergoing primary or revision surgery and computed binary logistic regression models. Logistic regression analysis revealed higher odds for revision surgery in CRS patients with older age (*p*-value < 0.001), male gender (*p*-value = 0.011), diagnosis of AERD (*p*-value = 0.005), and presence of asthma (*p*-value < 0.001) or allergies (*p*-value = 0.031). Confirming previous studies, we found that the factors of age, CRSwNP, AERD, allergies, and asthma are associated with revision ESS and identified surgical techniques that were predominantly used in revision cases.

## 1. Introduction

Chronic Rhinosinusitis (CRS) is a complex disease originating from an inflammatory process of the nose and paranasal sinuses [1]. When adequate medical treatment is ineffective, CRS frequently requires endoscopic sinus surgery (ESS). However, although ESS can improve quality of life (QOL)—which can last up to five years [2]—some CRS patients require multiple surgeries to achieve disease control. Inadequate disease control after ESS is associated with decreased QOL and complications [3]. Therefore, identifying factors associated with revision surgery in CRS patients is of great importance to improve patient counseling and identify those that might require multiple surgical procedures.

In a European international multicenter prevalence study, the prevalence of CRS was estimated at 10.9% [4], with a recently estimated prevalence of nasal polyps of 1.95% [5], making it a prevalent health problem. CRS diagnosis requires the presence of at least two out of four cardinal symptoms (i.e., nasal drainage, nasal obstruction, anosmia or hyposmia, and facial pain) for at least 12 successive weeks [6]. Clinically, CRS patients can be classified as CRS with nasal polyposis (CRSwNP) and CRS without nasal polyposis (CRSsNP). The clinical subtype of CRS affects recurrence rates, QOL, symptom control, and overall outcome [1,7,8,9]. CRSwNP can also be categorized into clinically relevant subtypes, such as allergic fungal rhinosinusitis (AFRS) or aspirin-exacerbated respiratory disease (AERD) [10].

Previous studies in CRS patients reported revision surgery rates of 11% after three years [11], 19% after five years [2], and 17% after ten years [1,12]. CRSwNP displays higher recurrence and revision rates than CRSsNP. Loftus et al. found an overall revision rate of 18.6% in patients with CRSwNP and even higher rates in those with AFRS (28.7%), AERD (27.2%), and asthma (22.6%) [13]. Koskinen et al. furthermore identified allergic rhinitis, corticosteroid treatment, previous CRS-surgery, and recurrent nasal polyps (NP) as factors associated with the need for revision ESS [14]. A large retrospective cohort study including data from 2005 to 2011 revealed that throughout the observational period, 6.65% of patients undergoing ESS required revision surgery with a mean time of 20.92 months between surgeries [15].

As surgical revision in CRS patients is associated with reduced QOL and potential risks and complications, it is important to further elucidate factors associated with revision surgeries. Therefore, this study aims to assess factors associated with revision ESS in CRS patients systematically.

## 2. Materials and Methods

This single-center study was approved by the Ethics committee of the Medical University of Vienna (EK-Nr.: 1736/2020) and conducted at the Department of Otorhinolaryngology at the Medical University of Vienna (MUV). All patients diagnosed with CRS based on consensus guidelines [1] undergoing ESS at the Medical University of Vienna (General Hospital of Vienna, Waehringer Guertel 18-20, 1090 Vienna, Austria) between 1 January 2012 and 31 December 2015 were retrospectively included. Data including demographics, CRS-specific variables, and surgical procedures were retrieved from the hospital’s patient record database.

Demographical data, including age at the time of the surgery (years), gender (male/female), smoking status (smoking/non-smoking), physician-diagnosed diabetes (yes/no), and previous nose/face traumata (yes/no), was retrieved from the hospital’s database. Patients were included twice if they underwent revision surgery within the above-mentioned study period.

Patients were divided into two groups, depending on whether the patients were undergoing primary or revision ESS. The CRS phenotype was classified based on the physician’s diagnosis: CRSsNP, CRSwNP, AFRS, and AERD. Furthermore, data concerning patients’ self-reported allergies (yes/no) and physician-diagnosed asthma (yes/no) was retrieved. Disease-specific characteristics included the number of the previous ESS (for revision cases) and the time since the last ESS (for revision cases, in months). Furthermore, the surgical method applied in each case was assessed based on the surgical protocol. Unlike patients undergoing “Full-house ESS”, who received surgical treatment of all paranasal sinuses, the patients undergoing “Limited ESS” only received surgical treatment of selected sinuses. The utilization of different surgical approaches (i.e., total ethmoidectomy, partial ethmoidectomy, maxillary antrostomy, medial maxillectomy, resection of the inferior nasal concha, and frontal sinus surgeries) was also retrieved.

Statistical analyses were performed using the statistical IBM software SPSS (Version 26.0 for MacOs, IBM Corp., Armonk, NY, USA) and Microsoft Excel (Redmond, WA, USA). The characteristics were listed for all surgeries performed throughout the four years of follow-up and divided by groups (i.e., revision or primary surgery). Continuous variables were presented as mean ± standard deviation (SD). Categorical variables were presented as absolute numbers (percentages).

To compare binary patient characteristics between primary and revision surgery patients, the χ^2^-test or Fisher’s exact test was applied. A two-tailed unpaired *t*-test for normally distributed continuous variables or the Wilcoxon rank test for continuous variables without normal distribution was used to facilitate comparisons between groups. Normal distribution was assessed by using histograms to display the data visually. We performed a binary logistic regression analysis with the outcome (revision: yes/no) to assess which factors were associated with revision surgery in our patient cohort. We entered the independent variables of age (years), gender (male/female), smoking status (smoking/non-smoking), diabetes (yes/no), AFRS (yes/no), AERD (yes/no), allergies (yes/no), asthma (yes/no) and CRSwNP (yes/no) to the binary logistic regression model in order to generate statistical estimates to calculate adjusted odds ratios (aOR) and 95% confidence intervals, thus controlling the potential influence of the above-mentioned confounders. For this study, the level of significance was set at 0.05.

## 3. Results

### 3.1. Descriptive Statistics

Throughout the observation period, 667 patients with a mean age of 41.5 years (standard deviation, SD = 15.5) underwent ESS at the MUV. The study population comprised 305 (45.7%) women and 362 (54.3%) men. Out of the 667 surgeries, 133 (19.9%) were revisions (i.e., the patient had already undergone one or more surgeries for CRS), and 534 (80.1%) cases were primary surgeries (i.e., no prior ESS had been performed). Twenty-one patients were included more than once, as they also required revision surgery throughout the observational period. The mean time since the last ESS was 6.6 months (standard deviation ± 5.7 months) in patients undergoing revision surgery. A total of 187 (28.0%) patients were classified as CRSsNP and 478 (71.7%) patients as CRSwNP. Fourteen (2.1%) patients were diagnosed with AFRS and 16 (2.4%) with AERD. Demographical characteristics are listed in Table 1, CRS-specific characteristics in Table 2.

### 3.2. Differences between Primary and Revision Surgery Patients

Patients requiring revision surgery were significantly older (mean age = 46 years) compared to primary cases (mean age = 40.4 years, *p*-value < 0.001). Among the patients requiring revision surgery were also significantly more men (63.2%, *p*-value = 0.022) (Table 1). Statistical analysis revealed that CRSwNP (*p*-value = 0.037), AERD (*p*-value < 0.001), allergies (*p*-value < 0.001) and asthma (*p*-value < 0.001) were diagnosed significantly more frequently in CRS patients undergoing revision surgery compared to primary cases (Table 2).

Concerning surgical methods used in primary and revision ESS, we found a significantly higher number of medial maxillectomy (*p*-value = 0.040), sphenoidotomy (*p*-value < 0.001) and frontal sinus surgeries (*p*-value < 0.001) among revision cases. Partial ethmoidectomy (*p*-value < 0.001) and maxillary antrostomy (*p*-value < 0.001) were performed significantly less frequently in revision cases compared to primary ESS.

### 3.3. Factors Associated with Revision Surgery in CRS Patients Undergoing ESS

As we identified demographics, such as gender and age to be significantly different in revision compared to primary cases, we wanted to know which other factors were associated with revision surgery. We therefore performed a binary logistic regression analysis with surgery as the binary outcome (primary/revision) and age (years), gender (male/female), smoking status (smoking/non-smoking), diabetes (yes/no), AFRS (yes/no), AERD (yes/no), allergies (yes/no), asthma (yes/no) and CRSwNP (yes/no) as explanatory variables (Table 3). Binary logistic regression analysis revealed that the factors of higher age (adjusted odds ratio, aOR, 1.025, 95% confidence interval, CI, 1.011–1.039) and male gender were associated with higher odds of revision surgery (aOR, 1.733, 95% CI, 1.132–2.654). Furthermore, the binary logistic regression model revealed that the diagnosis of AERD (aOR: 8.331, 95% CI: 1.874–37.040) was significantly associated with higher odds for revision surgery. The presence of asthma (aOR: 3.868; 95% CI: 1.844–8.111) and allergies (aOR: 1.622; 95% CI: 1.044–2.521) were also associated with revision surgery (Table 3).

## 4. Discussion

CRS is a complex and fairly common disease. Depending on the symptoms portrayed by the patients, several disease phenotypes of CRS (i.e., CRSwNP, CRSsNP, AERD, and AFRS) can be distinguished [10]. ESS is recommended in patients refractory to primary medical therapy to achieve adequate disease control. Nevertheless, disease recurrence is a problem with studies reporting revision rates of approximately 19% five years after ESS and even higher rates in patients diagnosed with CRSwNP [1]. Therefore, it is essential to elucidate further factors associated with disease recurrence and the need for revision ESS to improve patient counseling regarding prognosis and the expected outcome of surgery. In this study, we sought to identify different factors associated with revision ESS and disease recurrence. We found that the factors of higher age, male gender, CRSwNP, AERD, allergies, and physician-diagnosed asthma were significantly more frequent among CRS patients undergoing revision ESS. Furthermore, binary logistic regression analysis revealed higher odds for revision surgery in CRS patients with male gender, presence of AERD, allergies and asthma, and those higher in age. Unlike in patients undergoing primary ESS, the surgical procedures of medial maxillectomy, sphenoidotomy, and frontal sinus surgery were performed significantly more frequently in CRS patients undergoing revision surgery.

We found that older age was associated with higher odds for revision surgery. Our results are following previous studies, which also confirmed older age to be associated with the demand for revision ESS in CRS patients [16]. Interestingly, we found an association between male gender and higher odds of revision surgery, while the EPOS guidelines [1] state that female gender was associated with higher odds for revision surgery. However, the authors further explained that the factor of female gender was mainly associated with primary revision surgery, as the effect was not as noticeable in patients undergoing multiple revision surgeries [16]. Therefore, the discrepancy in our results might be explained by the fact that our study included many patients undergoing multiple revision surgeries and its cross-sectional nature.

Regarding the association between smoking and the need for revision ESS, a previous study over a 25-year-interval showed that active smoking was associated with a shorter time between primary and revision surgery in CRSwNP patients [17]. The authors explained that their findings might be caused by the deleterious effect of smoke on the sinonasal mucosa, facilitating faster polyp regrowth. Another study by Krzeski et al. also reported significantly more active smokers among CRS patients undergoing revision ESS. However, it did not show any effects of smoking on preoperative CT-scan-scores or pre/postoperative symptoms [18]. Our analysis, however, did not reveal any significant differences concerning the demand for revision surgery in comparison between smokers and non-smokers, which might be explained by the fact that we did not exclusively include CRSwNP patients and merely distinguished smokers and non-smokers, without clarifying whether patients had been smoking previously and how long.

In terms of disease phenotypes, the difference in outcome between patients diagnosed with CRSwNP and CRSsNP is well known. The current EPOS guidelines listed nasal polyps as highly associated with disease recurrence in CRS patients [1,16]. Although group comparisons confirmed previous findings, CRSwNP failed to reach significance in binary logistic regression analysis. These results might be explained by the single-center design of this study, which did not include patients who underwent revision surgery at a different hospital or patients who required revision surgery after the observational period.

Concerning the factors of allergies and asthma, we found a higher prevalence in CRS patients undergoing revision surgery. Furthermore, those two factors were also found to be associated with higher odds for revision ESS in binary logistic regression analysis. Our results follow previous studies, which also identified both allergy and asthma associated with revision surgery [1,16]. These findings can be explained by recent scientific findings linking type 2 inflammatory patterns in CRS patients to therapy resistance. When pathogens enter the mucosal barrier, three physiological responses can be defined: type 1 is targeted at viruses, type 2 at parasites, and type 3 at fungi and bacteria. CRS patients showcase different inflammatory endotypes, with a pure or mixed type 2 endotype being associated with therapy resistance and disease recurrence. Asthma and allergies are associated with type 2 inflammatory patterns and accentuate CRS’s type 2 inflammatory mechanisms [1]. Recent studies suggest that type 2 inflammation causes an imbalance of coagulative and fibrinolytic factors that contribute to the formation of a large fibrin mesh, which is suspected to be a primary driver in the (recurrent) formation process of nasal polyps [19].

As mentioned above, binary logistic regression analysis and group comparison revealed that AERD was associated with higher odds for revision surgery. Previous studies have shown that AERD is a predisposing factor for revision surgery and disease recurrence in CRS patients. AERD patients have been younger and require more sinus surgeries than CRSwNP patients [1,20]. These results might be explained by the extensive eosinophilic, type 2 inflammatory process caused by eicosanoid dysregulation [21], which can be observed in AERD patients and is associated with disease recurrence, as explained above. Those results further highlight the importance of comprehensive anamnesis and correct diagnoses to offer adequate treatment for different CRS disease phenotypes, thus avoiding lengthy and unsuccessful treatment.

ESS is often performed with other surgical procedures, such as septoplasty and/or turbinate surgery. In our study, 37.9% of all patients included underwent ESS with septoplasty. Group comparisons revealed that septoplasty was performed significantly less frequently in revisions, which is most likely caused by the fact that septoplasty is mostly performed in primary sinus surgery for anatomical and surgical reasons. Previous studies also demonstrated that revision rates in patients undergoing ESS and septoplasty were significantly lower than those undergoing ESS alone, which might be caused by narrower passageways impairing mucociliary clearance [22].

Although this study included a large cohort and revealed important factors associated with the need for revision surgery in CRS patients, there were some limiting factors. The single-center design certainly can be named as such a limiting factor. Furthermore, the cross-sectional design of this study only allowed us to perform analyses based on group comparisons. In contrast, a longitudinal study design would have allowed us to evaluate factors on an individual patient’s basis.

## 5. Conclusions

Confirming previous studies, we found that the factors of higher age, CRSwNP, AERD, allergies, and asthma were associated with revision ESS, confirming previous studies in CRS patients. Furthermore, we also found that medial maxillectomy, sphenoidotomy, and frontal sinus surgeries were performed significantly more often in revision cases. Our results further emphasize the importance of comprehensive history taking, endotyping and treatment planning in CRS patients.

## Figures and Tables

**Table 1:** Study participants’ demographical characteristics. Continuous variables are presented as mean ± SD (standard deviation), categorical variables as absolute number (percentage).

Demographics	All Cases (*n* = 667)	Primary Surgeries (*n* = 534)	Revisions (*n* = 133)	*p*-Value *
Age (years)	41.5 ± 15.5	40.4 ± 15.5	46 ± 14.5	**<0.001 ^1^**
Gender, female	305 (45.7%)	256 (47.9%)	49 (36.8%)	**0.022 ^2^**
Number of previous sinus surgeries	0.4 ± 1.2	-	2.0 ± 2.1	-
Smoking	180 (27.0%)	145 (27.2%)	35 (26.3%)	0.846 ^2^
Diabetes	23 (3.4%)	19 (3.6%)	4 (3.0%)	0.756 ^2^
Nose/face trauma	37 (5.5%)	29 (5.4%)	6 (6.0%)	0.792 ^2^

^1^ *t*-test, * *p*-value denotes group differences between primary and revision surgeries. ^2^ χ^2^-test, * bold values of *p* are significant.

**Table 2:** Chronic rhinosinusitis (CRS)-specific characteristics, such as CRS without nasal polyps (CRSsNP), CRS with nasal polys (CRSwNP), allergic fungal rhinosinusitis (AFRS) and aspirin-exacerbated respiratory disease (AERD) as absolute numbers and percentages.

CRS-Specific Characteristics	All Cases (*n* = 667)	Primary Surgeries (*n* = 534)	Revisions (*n* = 133)	*p*-Value *
CRSsNP	187 (28.0%)	159 (29.8%)	28 (21.1%)	**0.045 ^1^**
CRSwNP	478 (71.7%)	373 (69.9%)	105 (78.9%)	**0.037 ^1^**
AFRS	14 (2.1%)	11 (2.1%)	3 (2.3%)	0.551 ^2^
AERD	16 (2.4%)	3 (0.6%)	13 (9.8%)	**<0.001 ^2^**
Allergies	214 (32.1%)	154 (28.8%)	60 (45.1%)	**<0.001 ^1^**
Asthma	44 (6.6%)	19 (3.6%)	25 (18.8%)	**<0.001 ^1^**

^1^ χ^2^-test, * *p*-value denotes group differences between primary and revision surgeries. ^2^ Fisher’s exact test, * bold values of *p* are significant.

**Table 3:** Results of binary logistic regression analysis with the factors of age (years), gender (male/female), smoking status (smoking/non-smoking), diabetes (yes/no), allergic fungal rhinosinusitis, AFRS (yes/no), aspirin-exacerbated respiratory disease, AERD (yes/no), allergies (yes/no), asthma (yes/no) and polyps (yes/no) as explanatory variables in order to generate statistical estimates to calculate adjusted odds ratios (aOR) and 95% confidence intervals (CI).

Variables	Revision Surgery	
	aOR (95% CI)	*p*-Value *
Age (years)	1.025 (1.011–1.039)	**<0.000**
Gender		
Female	Reference	
Male	1.733 (1.132–2.654)	**0.011**
Smoking status		
Non-smoking	Reference	
Smoking	1.194 (0.757–1.882)	0.446
Diabetes		
No	Reference	
Yes	0.341 (0.089–1.300)	0.115
AFRS		
No	Reference	
Yes	0.975 (0.225–3.731)	0.971
AERD		
No	Reference	
Yes	8.331 (1.874–37.040)	**0.005**
Allergies		
No	Reference	
Yes	1.622 (1.044–2.521)	**0.031**
Asthma		
No	Reference	
Yes	3.868 (1.844–8.111)	**<0.000**
CRSwNP		
No	Reference	
Yes	1.316 (0.813–2.131)	0.263

* Bold values of *p* are significant.

## Data Availability

All data analyzed in this study can be obtained upon request to the corresponding author.
